# Linvoseltamab outcomes by frailty status in patients with relapsed/refractory multiple myeloma: a subgroup analysis of the LINKER-MM1 study

**DOI:** 10.1038/s41408-026-01572-1

**Published:** 2026-07-25

**Authors:** Hira Mian, Naresh Bumma, Joshua Richter, Mina Awad, Timothy J. Inocencio, C. Akunna Otele, Megan Seraphin, Tito Roccia, Glenn S. Kroog, Tanya M. Wildes

**Affiliations:** 1 McMaster University, Hamilton, ON Canada; 2 The Ohio State University Comprehensive Cancer Center, Columbus, OH USA; 3 Icahn School of Medicine at Mount Sinai, New York, NY USA; 4 Regeneron Pharmaceuticals Inc., Tarrytown, NY USA; 5 University of Nebraska Medical Center/Nebraska Medicine, Omaha, NE USA

**Keywords:** Myeloma, Haematological cancer

## Abstract

There is a paucity of data in frail patients with multiple myeloma (MM) receiving bispecific antibodies (bsAbs). In LINKER-MM1 (NCT03761108), linvoseltamab (human BCMA×CD3 bsAb) demonstrated high response rates and a generally manageable safety profile in relapsed/refractory (RR)MM. This LINKER-MM1 *post hoc* analysis evaluated outcomes of linvoseltamab 200 mg in patients categorized for frailty using the International Myeloma Working Group Frailty Proxy (IMWG-FP), Simplified Frailty Score (SFS), and patient-reported frailty phenotype (PRFP). As of July 23, 2024, 117 patients received linvoseltamab (median follow-up: 21.3 months). The percentage of frail patients varied by measure: IMWG-FP, 27.4%; SFS, 26.5%; and PRFP, 20.5% (8/4 patients were excluded due to missing IMWG-FP/PRFP scores, respectively). Objective response rates in non-frail and frail groups, respectively, were: IMWG-FP, 72.7% and 65.6%; SFS, 69.8% and 74.2%; PRFP, 73.0% and 62.5%. Incidence of Grade 3/4 treatment-emergent adverse events was consistent across non-frail and frail groups (IMWG-FP: 72.7% and 75.0%; SFS%: 73.3% and 74.2; PRFP: 74.2% and 70.8, respectively). Clinical outcomes were consistent in non-frail and frail groups, indicating a favorable risk–benefit profile for linvoseltamab in the frail patients with RRMM from LINKER-MM1. Continued evaluation of linvoseltamab in frail patients with RRMM in clinical trials and real-world settings is warranted.

## Introduction

Multiple myeloma (MM) is a progressive hematologic malignancy, predominantly affecting older people, that remains incurable despite advances in myeloma treatments [1, 2]. With a median age at diagnosis of 69 years [3], approximately one-third of patients with MM are aged >75 years and are often characterized as frail or having low fitness levels [4, 5]. Along with older age, comorbidities, and compromised organ functioning [6, 7], being frail is generally regarded as an indicator of poor prognosis in MM and has been associated with increased vulnerability to treatment toxicities [8–10].

Accurate identification of frailty is a prerequisite for assessing outcomes in frail patients; this is particularly important when evaluating new therapies, such as bispecific antibodies (bsAbs) [11–13]. In a systematic review, Mian et al. (2023) underscored a highly variable prevalence of frailty across MM clinical studies (17% to 74%); this wide variation in frailty classification, partially stemming from heterogeneity in the definition of frailty across different indices, may confound outcome assessment, cross-trial comparison, and, ultimately, decision-making [4, 8, 9]. Although validated frailty indices are available to categorize patient frailty status for the purpose of informing treatment decisions (Supplementary Fig. 1), their prospective use in clinical trials has been inconsistent due to operational-, cost-, and resource-intensive requirements [8]. This is the case for the International Myeloma Working Group (IMWG) Frailty Index, which is considered the gold-standard frailty assessment as it incorporates comprehensive patient-evaluated self-care and household management questionnaires (i.e., Katz Index of Independence in Activities of Daily Living and Lawton Instrumental Activity of Daily Living) [14], and for the Fried Frailty Phenotype index, which requires in-person physical performance assessments. To facilitate incorporation of frailty classification into clinical studies, simplified proxy measures have been developed to classify frail patients retrospectively by leveraging clinical data routinely collected within clinical trials. Measures such as the IMWG Frailty Proxy (IMWG-FP) [11] and the Simplified Frailty Score (SFS; previously known as Eastern Cooperative Oncology Group [ECOG] Frailty Proxy) [15], have been specifically developed from the IMWG index to classify frailty retrospectively; these measures utilize data from simple and practical EuroQoL questionnaires (i.e., EuroQoL five dimensions 3-level version and EuroQoL five dimensions 5-level version) and the ECOG performance status (PS), respectively, as substitutes for the Activities of Daily Living and Instrumental Activity of Daily Living assessments [11, 16]. Additionally, as a proxy for the Fried Frailty Phenotype index, the patient-reported frailty phenotype (PRFP) measure was shown to have adequate internal consistency, reliability, and structural validity for the retrospective evaluation of frailty [17]. Contrary to IMWG-FP and SFS, the PRFP measure excludes age as a variable, allowing patient classification based on self-reported frailty manifestations rather than chronological age [11, 17].

The emergence of bsAbs in recent years has represented a significant advancement in the treatment of MM [13]. BsAbs that target B-cell maturation antigen (BCMA) have demonstrated robust efficacy outcomes in patients with relapsed or refractory MM (RRMM) [2, 18–20]. Commonly reported treatment-emergent adverse events (TEAEs) associated with T-cell–engaging therapies such as bsAbs include cytokine release syndrome (CRS), neurotoxicity including immune effector cell-associated neurotoxicity syndrome (ICANS), and infections [2]. Based on the relatively recent introduction of these novel agents into clinical practice, there is a need to better characterize the risk–benefit profile of bsAbs, for which real-world data are currently emerging [21, 22]. Currently, there is a paucity of data on how frailty impacts efficacy and safety outcomes for patients treated with bsAbs [8]. Characterizing outcomes of bsAbs in frail patients could inform their use in this high-risk patient group and, most importantly, the need for any dose adjustments or concomitant medications to ensure appropriate maintenance of a favorable risk–benefit profile. In the absence of head-to-head studies, multiple frailty measures can be evaluated both in clinical trials, which have strict inclusion criteria, as well as in real-world settings.

Linvoseltamab, a fully human BCMA×cluster of differentiation 3 (CD3) bsAb, promotes cytolytic killing of BCMA-expressing MM plasma cells by CD3-positive effector T cells [23]. Based on the results from the phase 1/2 LINKER-MM1 study (NCT03761108) [24, 25], linvoseltamab was approved for adult patients with RRMM in the European Union and United States (accelerated approval) who have received ≥3 prior therapies or ≥4 prior lines of therapy, respectively, including a proteasome inhibitor (PI), an immunomodulatory drug (IMiD), and an anti-CD38 antibody. In this study, linvoseltamab 200 mg induced deep and durable responses with a generally manageable safety profile in patients with RRMM [2]. At a median follow-up of 21.3 months, linvoseltamab achieved a 71% overall response rate (ORR), a rate of complete response or better of 52%, and a median duration of response (DOR) of 29.4 months. The most frequent TEAEs were CRS (46%), neutropenia (44%), diarrhea (42%), anemia (40%), and cough (40%), with infections occurring in 75% of patients and the incidence of infections declining after six months of linvoseltamab treatment [26].

Here, we present a comprehensive *post hoc* analysis of the LINKER-MM1 study that explores the efficacy and safety of linvoseltamab in patients with RRMM based on the retrospective categorization of frailty by IMWG-FP [16], SFS [15], and PRFP [17] measures. For this analysis, IMWG-FP and SFS were selected based on standardized measures and common use, respectively, while PRFP was selected because of its objective, patient-centric assessment approach.

## Methods

### Patients and study design

LINKER-MM1 is an open-label, first-in-human, phase 1/2 clinical trial (NCT03761108). The study design and patient eligibility criteria of LINKER-MM1 have been previously published [2]. The study enrolled adult (≥18 years old) patients with active MM and response-evaluable disease as defined by the IMWG [27, 28], who had been exposed to ≥3 prior lines of therapy, including a PI, an IMiD, and an anti-CD38 antibody (triple-class exposed), or were double-class refractory (phase 1 patients refractory to both an IMiD and a PI) or triple-class refractory (phase 2 patients refractory to an IMiD, a PI, and an anti-CD38 antibody). Patients with an ECOG-PS ≥2 were excluded from the study. Key exclusion criteria were the presence of significant comorbidities, such as primary systemic light-chain amyloidosis, known central nervous system movement disorder or central nervous system involvement, or history of neurodegenerative condition. Patients with prior exposure to BCMA-directed antibody–drug conjugates were eligible for inclusion in LINKER-MM1, except for those previously treated with other BCMA-directed immunotherapies (chimeric antigen receptor T-cell therapies and bispecific T-cell engagers).

LINKER-MM1 was conducted in accordance with the principles of the Declaration of Helsinki. The study was approved by the institutional review board or independent ethics committee at each site, and all patients provided written informed consent prior to participation. All patients provided written informed consent before enrollment.

### Treatment

Linvoseltamab was administered intravenously using step-up dosing (5 mg in Week 1; 25 mg in Week 2) with premedication through the first full dose to mitigate the risk of infusion-related reactions and CRS. Patients received linvoseltamab 200 mg once weekly from Week 3 through Week 14 (phase 2) or Week 16 (phase 1), and then every 2 weeks until disease progression or study discontinuation. In the 200 mg cohort in phase 2, patients who achieved a very good partial response or better and had received ≥24 weeks of treatment transitioned from every 2 weeks to every 4 weeks dosing.

### Frailty assessments

Patients were categorized as non-frail or frail based on their baseline characteristics, by applying the three frailty measures defined *post hoc* within LINKER-MM1. The three frailty measures – IMWG-FP, SFS, and PRFP – and the components used for each are summarized in Supplementary Fig. 1.

Based on the IMWG Frailty Index [14], IMWG-FP was calculated using age (≤75 [score 0], 76–80 [score 1], *>*80 years [score 2]), Charlson Comorbidity Index (CCI; ≤1 [score 0], ≥2 [score 1]), and EQ-5D-3L scores on self-care (no problems [score 0], moderate/severe problems [score 1]) and usual activities (no problems [score 0], moderate/severe problems [score 1]). We used the same dichotomization thresholds in the present study as proposed by Facon et al. 2015 [11, 16]. For the purposes of our analysis, patients were categorized as non-frail (summed scores of 0–1) or frail (summed scores of ≥2); the original IMWG-FP comprised three categories: frail, intermediate fitness, or fit [15, 16].

Based on the IMWG Frailty Index [14], SFS was calculated using age ( ≤75 [score 0], 76–80 [score 1], *>*80 years [score 2]), CCI (≤1 [score 0], ≥2 [score 1]), and ECOG-PS (0 [score 0], 1 [score 1], ≥2 [score 2]). We used the same thresholds in the present study as proposed by Facon et al. 2020, and categorized patients into non-frail (summed scores of 0–1) or frail (summed scores of ≥2) groups [15].

PRFP was calculated using patient responses to European Organization for Research and Treatment of Cancer Quality of Life 30-item Questionnaire (EORTC QLQ-C30) items that measured concepts similar or identical to the original FFP [17, 29]. We used the same dichotomization thresholds as proposed by Murugappan et al. 2023, 2024 [11, 17]. For the purposes of our analysis, we categorized patients as non-frail (summed scores of 0–2) or frail (summed scores of ≥3) contrarily to the original PRFP comprising three categories: frail, pre-frail, or fit.

### Outcomes

The phase 2 primary endpoint of LINKER-MM1 was ORR of linvoseltamab, which was assessed by an Independent Review Committee (IRC) per IMWG criteria [26, 28]. Additional outcomes included DOR, progression-free survival (PFS) as assessed by both an IRC and the investigator, overall survival (OS), safety, and tolerability.

### Statistical analysis

The present *post hoc* analysis was performed with data from the phase 1/2 LINKER-MM1 study of patients who received the full 200 mg dose of linvoseltamab. Patients who received at least one full 200 mg dose of linvoseltamab were included in the safety and efficacy analysis. Only patients with non-missing scores were categorized by each frailty measure. The data cut-off was July 23, 2024, for the efficacy and safety analyses. The Clopper–Pearson exact method was used to generate 95% confidence intervals (CIs) for ORR. DOR, PFS, and OS were estimated using Kaplan–Meier (KM) analysis. Descriptive statistics were used to summarize categorical and continuous data, including baseline characteristics and patient-reported outcome (PRO) data, as well as the numbers and proportions of patients categorized as frail and non-frail by the IMWG-FP, SFS, and PRFP measures.

## Results

### Patients

At data cut-off, 117 patients received linvoseltamab 200 mg, with a median follow-up of 21.3 months. Patients were categorized as frail or non-frail as per the three frailty measures, IMWG-FP, SFS, and PRFP.

**Fig. 1:**
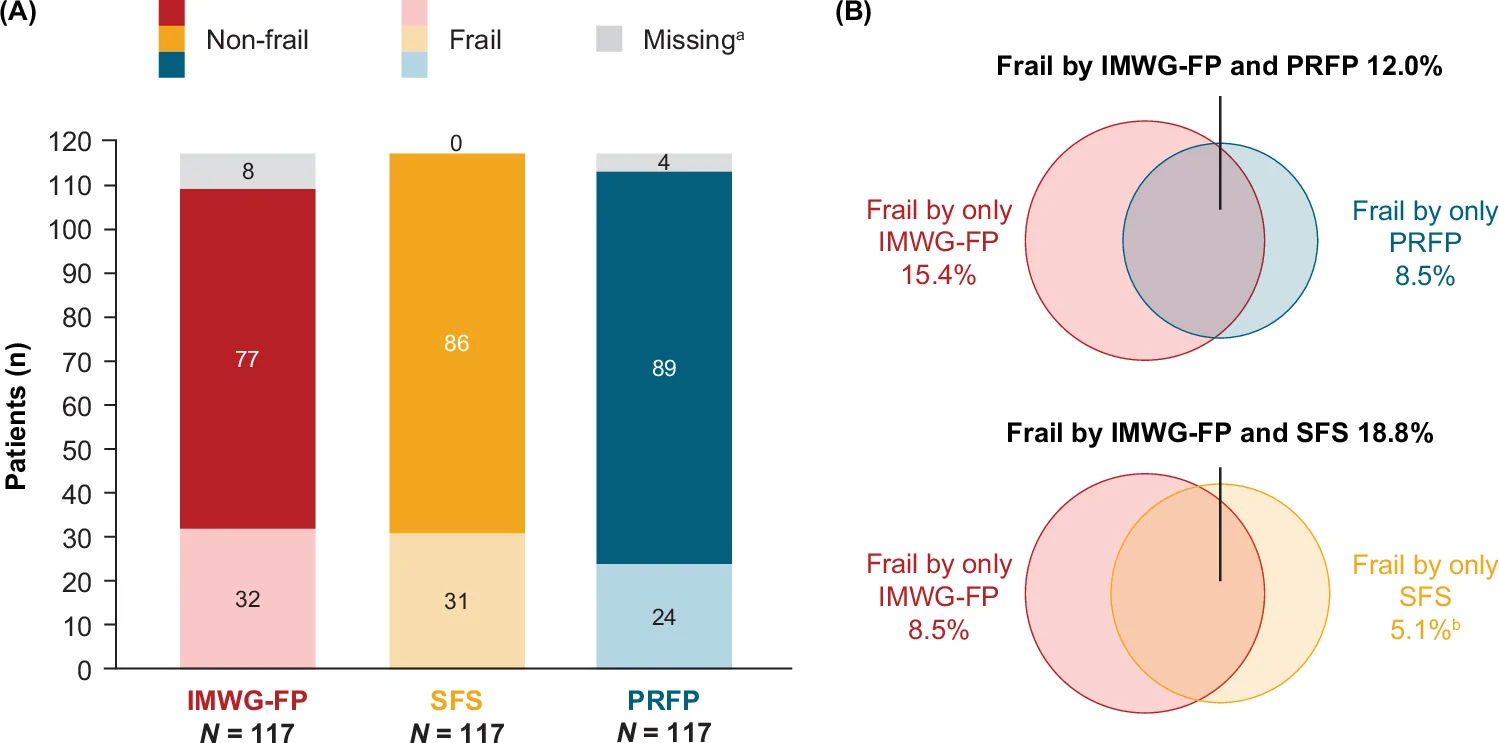
Patients by frailty measure. Categorization of patients by each frailty measure (**A**) and concordance across frailty measures (**B**). ^a^IMWG-FP, missing EQ-5D-3L data in eight patients; PRFP, missing EORTC QLQ-C30 data in four patients; ^b^Missing data in three patients. SFS, Simplified EORTC QLQ-C30, European Organization for Research and Treatment of Cancer Quality of Life 30-item Questionnaire; EQ-5D-3L, EuroQol five dimensions 3-level version; EQ-5D-5L, EuroQol five dimensions 5-level version; IMWG-FP, International Myeloma Working Group Frailty Proxy; PRFP, patient-reported frailty phenotype; SFS, Simplified Frailty Score.

Per IMWG-FP, 32 patients (27.4%) were categorized as frail and 77 (65.8%) as non-frail (8 patients [6.8%] had a missing/unreported baseline EQ-5D-3L score and therefore could not be categorized). Per SFS, 31 (26.5%) patients were categorized as frail and 86 (73.5%) as non-frail. Finally, per PRFP, 24 patients (20.5%) were categorized as frail and 89 (76.1%) as non-frail (4 patients [3.4%] missing). The proportion of patients who were categorized as frail by both IMWG-FP and PRFP measures was 12.0%, while the proportion of patients categorized as frail by both IMWG-FP and SFS was 18.8% (Fig. 1).

#### Baseline characteristics

Baseline characteristics varied between non-frail and frail groups depending on the frailty measure used (Table 1).

**Table 1:** Baseline and disease characteristics.

Characteristics	IMWG-FP		SFS		PRFP	
	Non-frail (*n* = 77)	Frail (*n* = 32)	Non-frail (*n* = 86)	Frail (*n* = 31)	Non-frail (*n* = 89)	Frail (*n* = 24)
**Age**						
Median years, (range)	65 (37–80)	75 (44–91)	66 (37–80)	77 (56–91)	71 (42–83)	68 (37–91)
≤75 years, *n* (%)	72 (93.5)	17 (53.1)	82 (95.3)	12 (38.7)	72 (80.9)	19 (79.2)
>75 years, *n* (%)	5 (6.5)	15 (46.9)	4 (4.7)	19 (61.3)	17 (19.1)	5 (20.8)
**Female**, *n* **(%)**	35 (45.5)	15 (46.9)	40 (46.5)	13 (41.9)	36 (40.4)	15 (62.5)
**Race**, *n* **(%)**						
White	57 (74.0)	19 (59.4)	65 (75.6)	18 (58.1)	65 (73.0)	15 (62.5)
Asian	5 (6.5)	5 (15.6)	6 (7.0)	4 (12.9)	7 (7.9)	3 (12.5)
Black/African American	12 (15.6)	7 (21.9)	12 (14.0)	8 (25.8)	15 (16.9)	4 (16.7)
Other/not reported	3 (3.9)	1 (3.1)	3 (3.5)	1 (3.2)	2 (2.2)	2 (8.3)
**ECOG-PS**, *n* **(%)**						
0	26 (33.8)	3 (9.4)	31 (36.0)	1 (3.2)	27 (30.3)	3 (12.5)
1	51 (66.2)	29 (90.6)	55 (64.0)	30 (96.8)	62 (69.7)	21 (87.5)
**ISS**, *n* **(%)**						
I	37 (48.1)	9 (28.1)	41 (47.7)	8 (25.8)	36 (40.4)	12 (50.0)
II	28 (36.4)	11 (34.4)	30 (34.9)	11 (35.5)	32 (36.0)	8 (33.3)
III	8 (10.4)	11 (34.4)	10 (11.6)	11 (35.5)	16 (18.0)	4 (16.7)
Missing	4 (5.2)	1 (3.1)	5 (5.8)	1 (3.2)	5 (5.6)	0
**R-ISS**, *n* **(%)**						
I	22 (28.6)	4 (12.5)	25 (29.1)	3 (9.7)	21 (23.6)	6 (25.0)
II	43 (55.8)	19 (59.4)	46 (53.5)	19 (61.3)	50 (56.2)	14 (58.3)
III	7 (9.1)	8 (25.0)	9 (10.5)	8 (25.8)	12 (13.5)	4 (16.7)
Missing	5 (6.5)	1 (3.1)	6 (7.0)	1 (3.2)	6 (6.7)	0
**Cytogenetic risk**, *n* **(%)**						
High	31 (40.3)	12 (37.5)	33 (38.4)	13 (41.9)	33 (37.1)	11 (45.8)
Standard	46 (59.7)	20 (62.5)	53 (61.6)	18 (58.1)	56 (62.9)	13 (54.2)
**BMPC**, *n* **(%)**						
≥60%	13 (16.9)	8 (25.0)	17 (19.8)	6 (19.4)	14 (15.7)	9 (37.5)
<60%	48 (62.3)	18 (56.3)	51 (59.3)	20 (64.5)	56 (62.9)	12 (50.0)
Missing	14 (18.2)	6 (18.8)	16 (18.6)	5 (16.1)	17 (19.1)	3 (12.5)
**EMP per IRC**, *n* **(%)**						
With	6 (7.8)	10 (31.3)	13 (15.1)	6 (19.4)	12 (13.5)	6 (25.0)
Without	71 (92.2)	22 (68.8)	73 (84.9)	25 (80.6)	77 (86.5)	18 (75.0)
**sBCMA**, *n* **(%)**						
≥400 ng/mL	28 (36.4)	21 (65.6)	35 (40.7)	18 (58.1)	37 (41.6)	13 (54.2)
<400 ng/mL	46 (59.7)	9 (28.1)	48 (55.8)	11 (35.5)	47 (52.8)	11 (45.8)
Missing	3 (3.9)	2 (6.3)	3 (3.5)	2 (6.5)	5 (5.6)	0
**Median CrCl (range), mL/min**	78.2 (33.2–174.5)	58.8 (25.5–175.7)	76.9 (33.4–174.5)	57.5 (25.5–175.7)	70.9 (25.5–175.7)	78.5 (25.7–174.5)
**Median LDH (range), IU/L**	193.5 (115–576)	197.0 (116–953)	192.0 (115–953)	207.0 (121–922)	194.5 (115–953)	204.5 (116–922)
**Median number of prior lines, (range)**	5 (2–16)	4.5 (3–10)	5 (2–16)	5 (3–13)	5 (2–13)	4 (3–16)
**Exposure status**, *n* **(%)**						
Triple-exposed	77 (100)	32 (100)	86 (100)	31 (100)	89 (100)	24 (100)
Quad-exposed	72 (93.5)	32 (100)	81 (94.2)	31 (100)	85 (95.5)	23 (95.8)
Penta-exposed	59 (76.6)	23 (71.9)	66 (76.7)	24 (77.4)	67 (75.3)	19 (79.2)
**Refractory status**, *n* **(%)**						
Triple-refractory	62 (80.5)	27 (84.4)	70 (81.4)	26 (83.9)	72 (80.9)	21 (87.5)
Quad-refractory	49 (63.6)	22 (68.8)	55 (64.0)	23 (74.2)	58 (65.2)	17 (70.8)
Penta-refractory	21 (27.3)	10 (31.3)	24 (27.9)	10 (32.3)	27 (30.3)	7 (29.2)
Refractory to last line	65 (84.4)	28 (87.5)	75 (87.2)	26 (83.9)	78 (87.6)	19 (79.2)

*BMPC* bone marrow plasma cells, *CrCI* creatinine clearance, *ECOG-PS* Eastern Cooperative Oncology Group performance status, *EMP* extramedullary plasmacytoma, *IMWG-FP* International Myeloma Working Group Frailty Proxy, *IRC* Independent Review Committee, *ISS* International Staging System, *IU* International Unit, *LDH* lactate dehydrogenase, *PRFP* patient-reported frailty phenotype, *R-ISS* revised International Staging System, *sBCMA* soluble B-cell maturation antigen, *SFS* Simplified Frailty Score.

Across IMWG-FP and SFS measures, there were no differences in sex or prior treatment history by frailty status. Patients categorized as frail by IMWG-FP and SFS also tended to be older than those categorized as non-frail by the same measure. Compared with the non-frail group, proportions of patients with baseline International Staging System (ISS) or revised ISS (R-ISS) stage III myeloma and patients with extramedullary plasmacytomas (EMPs) were higher in the frail group by IMWG-FP and SFS. A higher proportion of patients categorized by IMWG-FP as frail had a baseline soluble BCMA (sBCMA) concentration of ≥400 ng/mL, compared with those categorized as non-frail. A similar pattern was observed when comparing patients categorized as frail versus non-frail by SFS. The proportions of patients with high cytogenetic risk were comparable between patients categorized as frail or non-frail by SFS. Differences in proportions between patients categorized as frail versus non-frail with EMP and sBCMA concentrations ≥400 ng/mL were less marked with SFS versus IMWG-FP, but otherwise the magnitudes of differences were similar with either of these measures.

Comparison of patients categorized as frail versus non-frail by PRFP yielded some different findings to IMWG-FP and SFS. Firstly, age distributions did not differ by frailty assessed by PRFP, while the proportion of females was higher among patients categorized as frail. The increases in percentages of patients with ECOG-PS 1 and R-ISS stage III disease in the frail versus non-frail group were less marked with PRFP than with IMWG-FP or SFS. Similarly, with PRFP (unlike the other two measures), the percentage with ISS stage III MM was comparable between the frail and non-frail groups. Higher proportions of patients categorized by PRFP as frail had high cytogenetic risk and ≥60% bone marrow plasma cells (BMPC), compared with those categorized as non-frail.

#### Baseline PRO scores

**Fig. 2:**
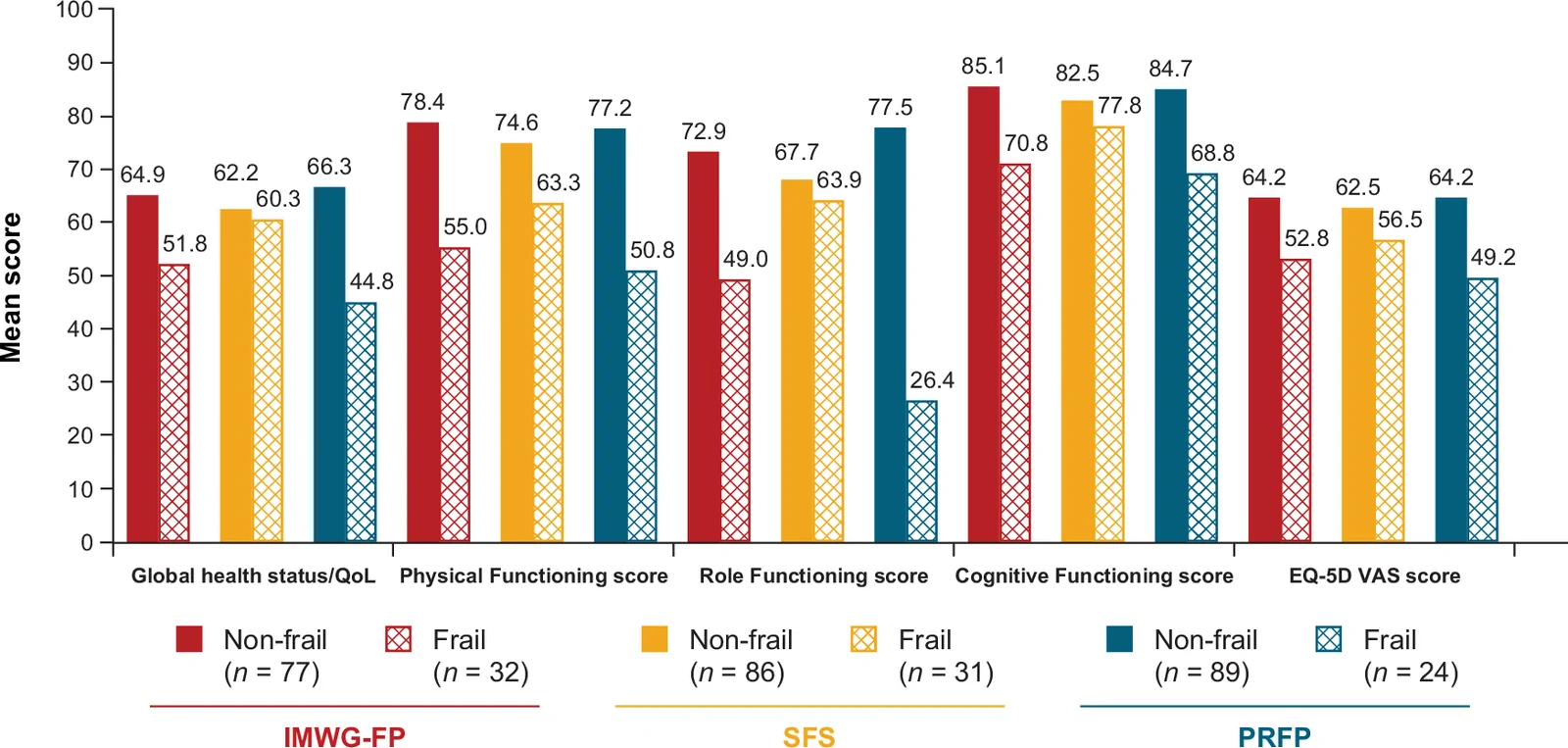
Baseline PRO scores. EQ-5D, EuroQol 5-Dimension; IMWG-FP, International Myeloma Working Group Frailty Proxy; PRFP, patient-reported frailty phenotype; PRO, patient-reported outcome; QOL, quality of life; SFS, Simplified Frailty Score; VAS, visual analog scale.

Lower baseline health-related quality of life (HRQOL) scores were observed in patients categorized as frail versus non-frail across all three frailty measures (Fig. 2), especially by IMWG-FP and PRFP. Overall, the largest difference between groups was observed for the ‘Role functioning’ domain, indicating decreased ability to engage in usual activities and roles (e.g., work, social) among frail patients.

### Efficacy

#### Median duration of follow-up

In the LINKER-MM1 population, the median duration of follow-up at data cut-off was 21.3 months (range 0.2–45.0 months). When comparing SFS and PRFP measures, median duration of follow-up was similar across non-frail (21.6 months [1.0–45.0]; 21.4 months [0.2–45.0]) and frail (20.3 months [0.2–39.1]; 21.3 months [0.3–41.7]) groups. A different pattern was apparent when using IMWG-FP, for which a median (range) duration of follow-up of 21.9 months (1.0–45.0) was found for non-frail versus 16.0 months (0.2–39.1) for frail groups.

#### Objective response rate

**Fig. 3:**
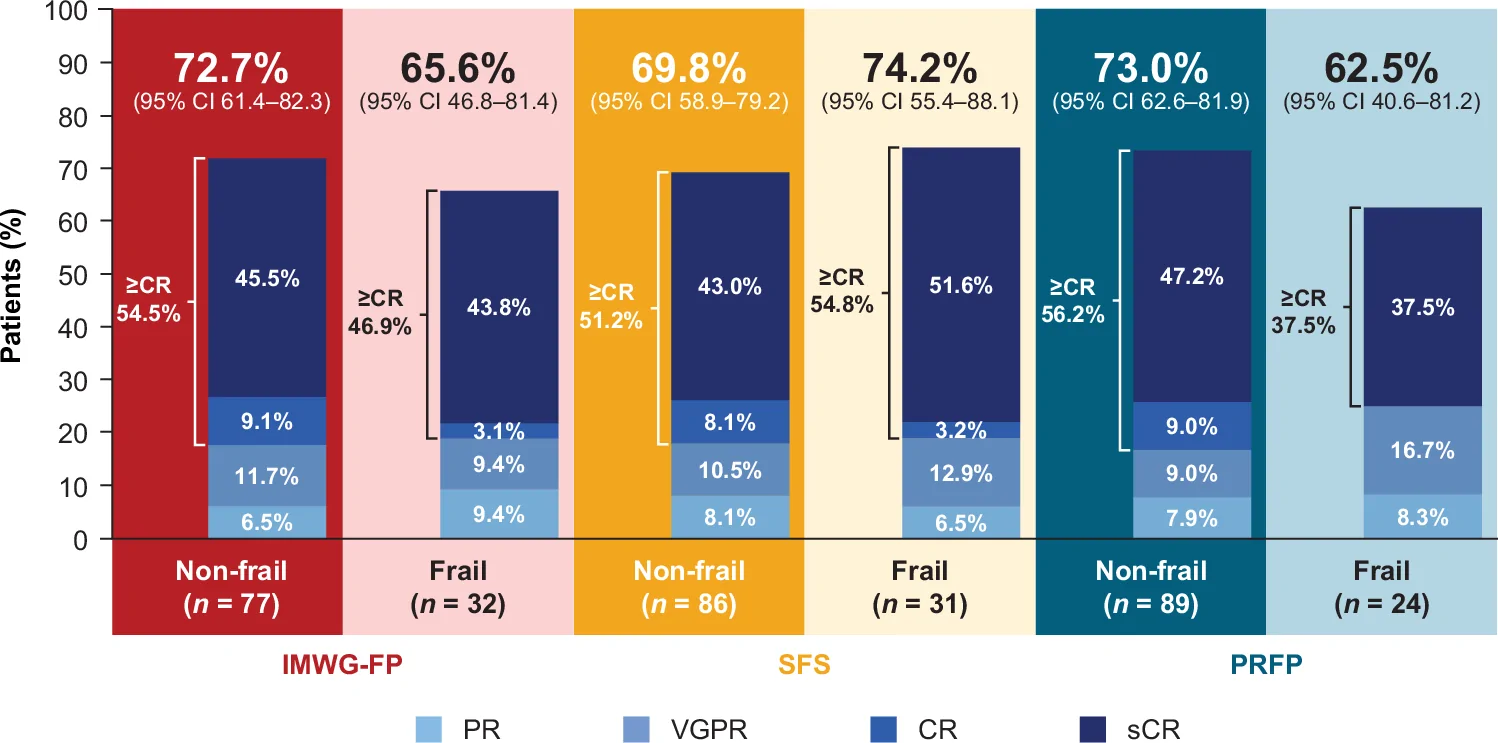
ORR in non-frail and frail groups by frailty measure. ORR was assessed by an IRC per IMWG criteria. CI, confidence interval; CR, complete response; IMWG, International Myeloma Working Group; IMWG-FP, International Myeloma Working Group Frailty Proxy; IRC, Independent Review Committee; ORR, objective response rate; PR, partial response; PRFP, patient-reported frailty phenotype; sCR, stringent complete response; SFS, Simplified Frailty Score; VGPR, very good partial response.

Generally, ORR and CR rates were similar between non-frail and frail groups across all three measures (Fig. 3). Per IMWG-FP and SFS, comparable rates of complete response (CR) or better were achieved by both non-frail (54.5% and 51.2%) and frail (46.9% and 54.8%) groups. A substantially lower rate of CR or better was observed in frail (37.5%) versus non-frail (56.2%) groups by PRFP.

#### Duration of response

**Fig. 4:**
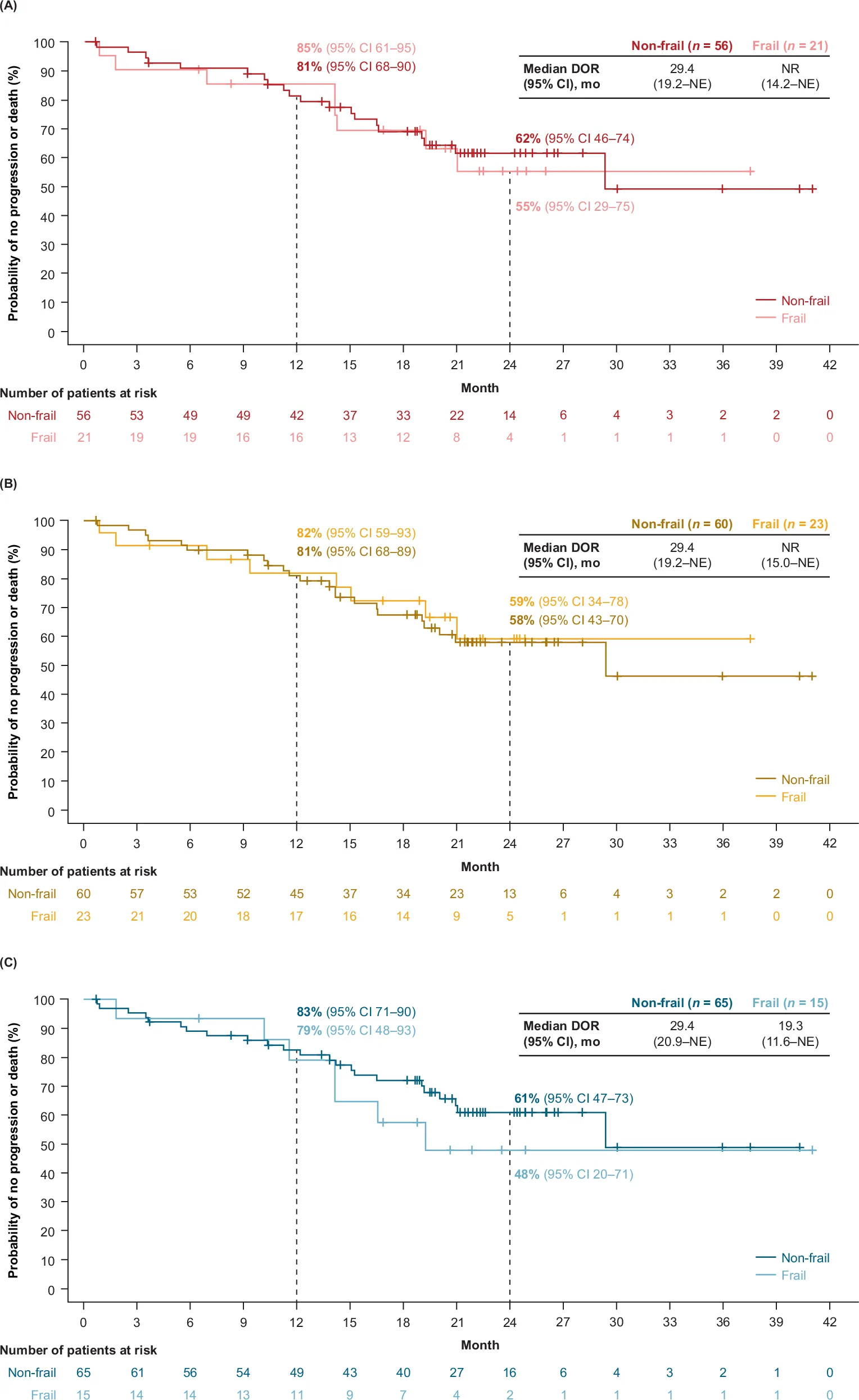
DOR in non-frail and frail groups by frailty measure. KM analysis of DOR among non-frail and frail groups defined by IMWG-FP (**A**), SFS (**B**), and PRFP (**C**). DOR was assessed by an IRC per IMWG criteria and the investigator. CI, confidence interval; DOR, duration of response; IMWG, International Myeloma Working Group; IMWG-FP, International Myeloma Working Group Frailty Proxy; IRC, Independent Review Committee; KM, Kaplan–Meier; mo, month; NE, not evaluable; NR, not reached; PRFP, patient-reported frailty phenotype; SFS, Simplified Frailty Score.

Linvoseltamab induced similar DOR in all non-frail groups, as defined by the three measures (Fig. 4). While median DOR was not reached in frail groups by IMWG-FP and SFS (Fig. 4A/B), patients categorized as frail by PRFP appeared to have a faster rate of disease progression or death (median DOR: 19.3 months [95% CI 11.6–not evaluable; NE]) than the non-frail group (median DOR: 29.4 months [95% CI 20.9–NE]) (Fig. 4C). Overall, the estimated probabilities of sustaining a response to linvoseltamab at month 24 in non-frail and frail groups were similar by SFS (58% and 59%) and modestly different by IMWG-FP (62% and 55%) and PRFP (61% and 48%).

#### Progression-free survival and overall survival

**Fig. 5:**
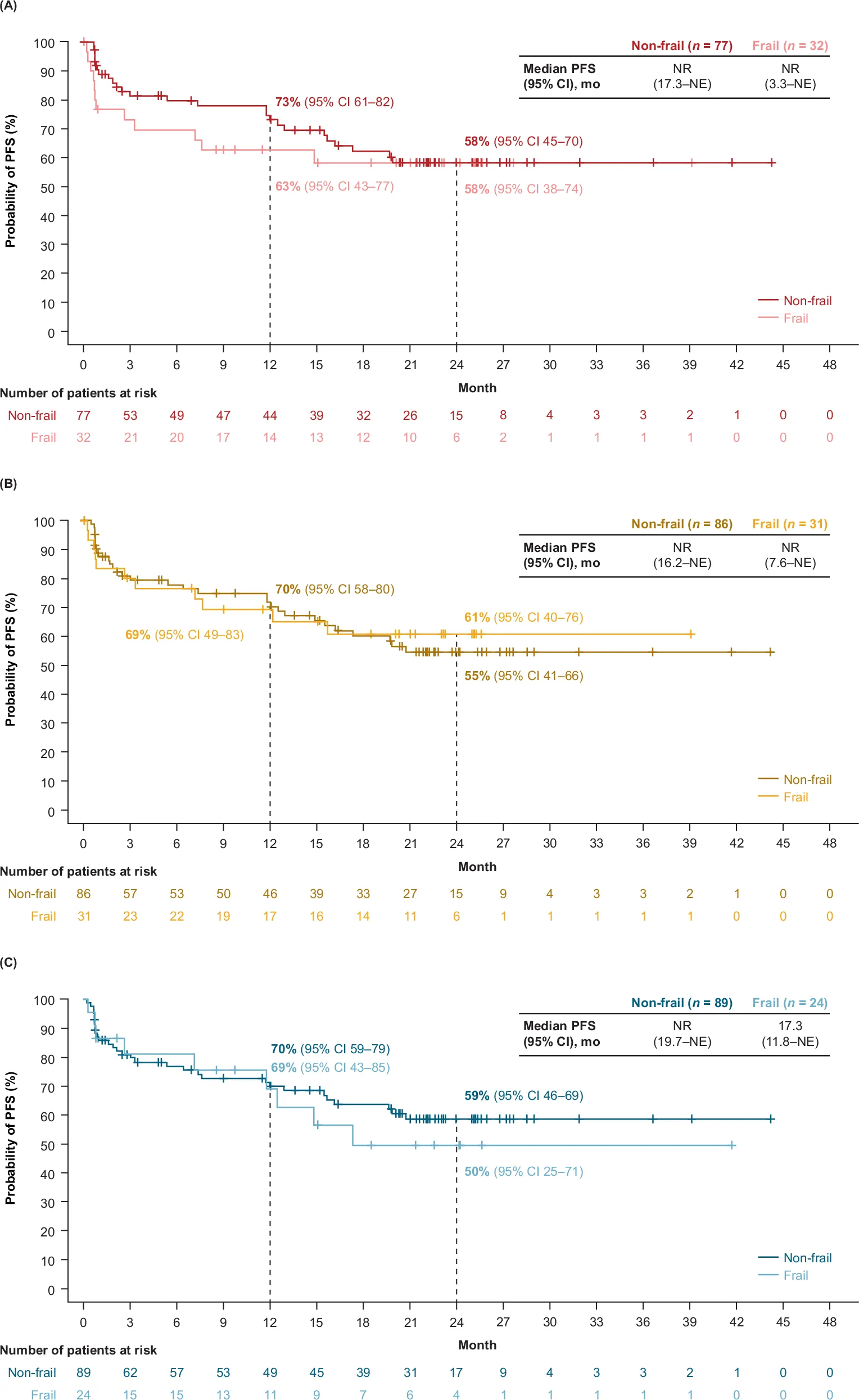
PFS in non-frail and frail groups by frailty measure. KM analysis of PFS in non-frail and frail groups defined by IMWG-FP (**A**), SFS (**B**), and PRFP (**C**). CIs are not presented on the graphs, given the substantial overlap between the frail and non-frail groups due to the small number of patients in each group. PFS was assessed by an IRC per IMWG criteria and the investigator. CI, confidence interval; IMWG, International Myeloma Working Group; IMWG-FP, International Myeloma Working Group Frailty Proxy; IRC, Independent Review Committee; KM, Kaplan–Meier; mo, month; NE, not evaluable; NR, not reached; PRFP, patient-reported frailty phenotype; PFS, progression-free survival; SFS, Simplified Frailty Score.

Overall, 12- and 24-month PFS were generally similar between non-frail and frail groups across all frailty measures (Fig. 5); slightly lower rates were observed in frail versus non-frail groups for the 12-month PFS per IMWG-FP (frail: 63%; non-frail: 73%; Fig. 5A) and for the 24-month PFS per PRFP (frail: 50%; non-frail: 59%; Fig. 5C). While median PFS was not reached by both non-frail and frail groups per IMWG-FP and SFS (Fig. 5A/B) and by the non-frail group per PRFP, a median PFS of 17.3 months (95% CI 11.8–NE) were reported in the frail group per PRFP (Fig. 5C).

**Fig. 6:**
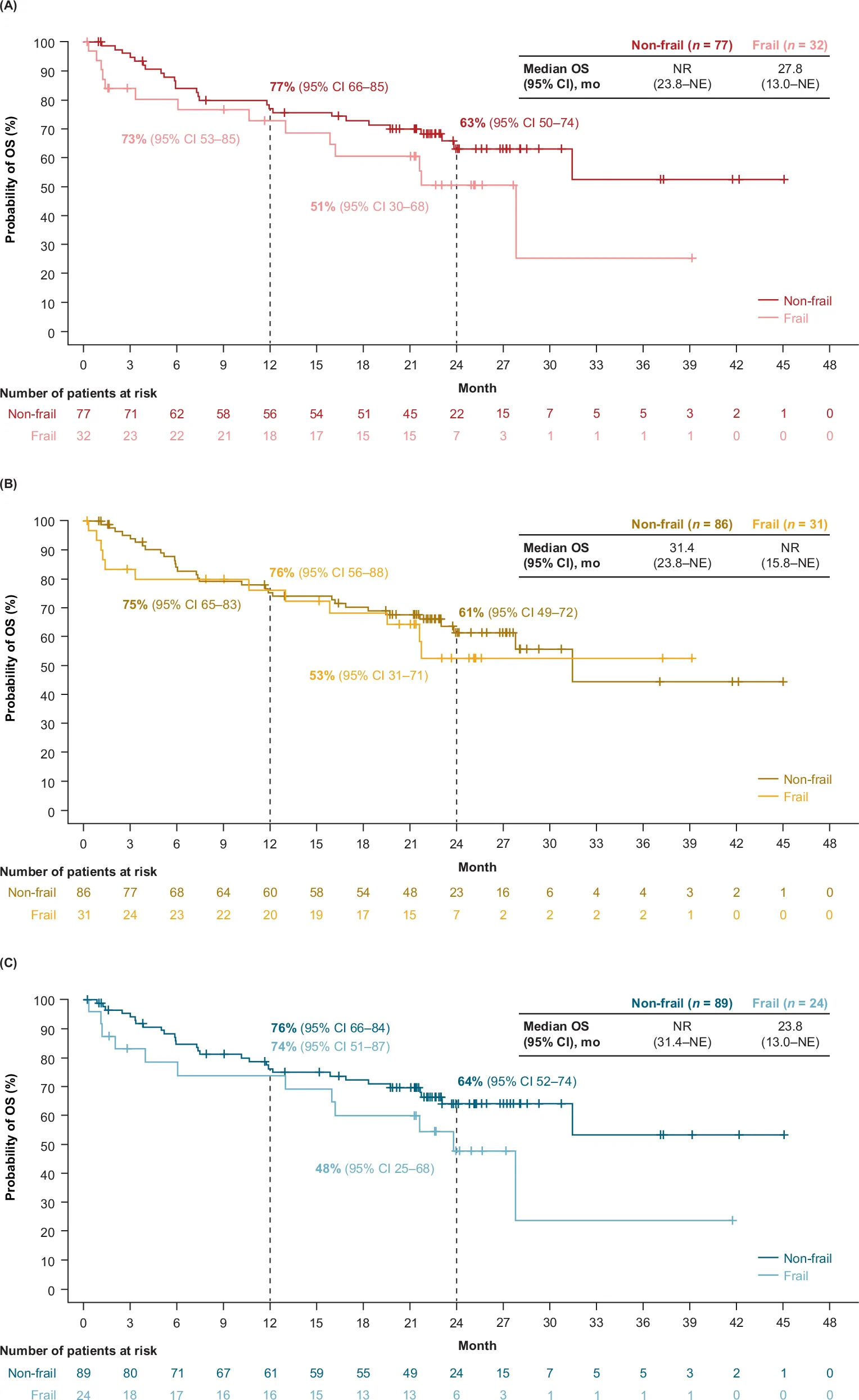
OS in non-frail and frail groups by frailty measure. KM analysis of OS in non-frail and frail groups defined by IMWG-FP (**A**), SFS (**B**), and PRFP (**C**). CIs are not presented given the substantial overlap between the non-frail and frail groups and the small number of patients in each group. CI, confidence interval; IMWG-FP, International Myeloma Working Group Frailty Proxy; IRC, Independent Review Committee; KM, Kaplan–Meier; mo, month; NE, not evaluable; NR, not reached; OS, overall survival; PRFP, patient-reported frailty phenotype; SFS, Simplified Frailty Score.

While similar 12-month OS were observed in non-frail (75–77%) and frail (73–76%) groups across all frailty measures (Fig. 6), for the 24-month OS, slightly lower rates were observed in frail versus non-frail groups per IMWG-FP (frail: 51%; non-frail: 63%; Fig. 6A) and per SFS (frail: 53%; non-frail: 61%; Fig. 6B), with the largest difference being observed per PRFP (frail: 48%; non-frail: 64%; Fig. 6C). Similar median OS was reported in the frail group per IMWG-FP (27.8 months [95% CI 13.0–NE]) and per PRFP (23.8 months [95% CI 13.0–NE]) (Fig. 6A/C), while it was not reached by the frail group per SFS (95% CI 15.8–NE; Fig. 6B).

### Safety

Across the three frailty measures, frail groups had a lower duration of treatment exposure compared with non-frail groups (Table 2).

**Table 2:** Treatment exposure and adverse events.

	IMWG-FP		SFS		PRFP	
	Non-frail (*n* = 77)	Frail (*n* = 32)	Non-frail (*n* = 86)	Frail (*n* = 31)	Non-frail (*n* = 89)	Frail (*n* = 24)
**Median treatment exposure (range), weeks**	62.1 (2–194)	39.4 (1–170)	54.1 (2–194)	48.0 (1–170)	61.0 (2–194)	49.9 (1–160)
**TEAEs**, *n* **patients (%)**						
Any grade	77 (100)	32 (100)	86 (100)	31 (100)	89 (100)	24 (100)
Grade 3/4	56 (72.7)	24 (75.0)	63 (73.3)	23 (74.2)	66 (74.2)	17 (70.8)
Any treatment-related	69 (89.6)	27 (84.4)	78 (90.7)	25 (80.6)	79 (88.8)	20 (83.3)
Serious	60 (77.9)	25 (78.1)	66 (76.7)	25 (80.6)	68 (76.4)	19 (79.2)
Leading to treatment discontinuation	18 (23.4)	4 (12.5)	20 (23.3)	4 (12.9)	17 (19.1)	5 (20.8)
Leading to dose interruption/delay	58 (75.3)	25 (78.1)	63 (73.3)	27 (87.1)	69 (77.5)	17 (70.8)
Leading to dose reduction	13 (16.9)	6 (18.8)	14 (16.3)	7 (22.6)	16 (18.0)	5 (20.8)
TEAE resulting in death	9 (11.7)	7 (21.9)	11 (12.8)	6 (19.4)	11 (12.4)	5 (20.8)
TEAE other than infection^a^	0	4 (12.5)	1 (1.2)	3 (9.7)	3 (3.4)	1 (4.2)
**Deaths up to 30 days from the end of treatment**, *n* **(%)**						
Any deaths	4 (5.2)	4 (12.5)	5 (5.8)	4 (12.9)	6 (6.7)	2 (8.3)
Due to adverse event	3 (3.9)	3 (9.4)	4 (4.7)	3 (9.7)	5 (5.6)	1 (4.2)
Due to progressive or recurring disease	1 (1.3)	1 (3.1)	1 (1.2)	1 (3.2)	1 (1.1)	1 (4.2)
**CRS**, *n* **patients (%)**						
Any grade	35 (45.5)	15 (46.9)	40 (46.5)	14 (45.2)	37 (41.6)	14 (58.3)
Grade 3/4	1 (1.3)	0	1 (1.2)	0	0	1 (4.2)
Serious	22 (28.6)	7 (21.9)	26 (30.2)	6 (19.4)	22 (24.7)	8 (33.3)
Leading to treatment discontinuation	0	0	0	0	0	0
Leading to dose interruption/delay	4 (5.2)	3 (9.4)	6 (7.0)	1 (3.2)	3 (3.4)	4 (16.7)
Leading to dose reduction	2 (2.6)	3 (9.4)	3 (3.5)	2 (6.5)	2 (2.2)	3 (12.5)
CRS resulting in death	0	0	0	0	0	0
**ICANS**, *n* **patients (%)**						
Any grade	4 (5.2)	4 (12.5)	6 (7.0)	3 (9.7)	7 (7.9)	2 (8.3)
Grade 3/4	1 (1.3)	2 (6.3)	2 (2.3)	1 (3.2)	1 (1.1)	2 (8.3)
Serious	2 (2.6)	3 (9.4)	3 (3.5)	2 (6.5)	3 (3.4)	2 (8.3)
Leading to treatment discontinuation	1 (1.3)	0	1 (1.2)	0	1 (1.1)	0
Leading to dose interruption/delay	1 (1.3)	1 (3.1)	1 (1.2)	1 (3.2)	1 (1.1)	1 (4.2)
Leading to dose reduction	1 (1.3)	0	1 (1.2)	0	1 (1.1)	0
ICANS resulting in death	0	0	0	0	0	0
**Infections**, *n* **patients (%)**						
Any grade	57 (74.0)	24 (75.0)	63 (73.3)	25 (80.6)	64 (71.9)	20 (83.3)
Grade 3/4	27 (35.1)	13 (40.6)	31 (36.0)	12 (38.7)	32 (36.0)	10 (41.7)
Serious	34 (44.2)	15 (46.9)	38 (44.2)	15 (48.4)	38 (42.7)	13 (54.2)
Leading to treatment discontinuation	10 (13.0)	1 (3.1)	11 (12.8)	1 (3.2)	8 (9.0)	3 (12.5)
Leading to dose interruption/delay	36 (46.8)	15 (46.9)	38 (44.2)	18 (58.1)	42 (47.2)	12 (50.0)
Leading to dose reduction	4 (5.2)	2 (6.3)	5 (5.8)	3 (9.7)	7 (7.9)	1 (4.2)
Infection resulting in death^b^	9 (11.7)	3 (9.4)	10 (11.6)	3 (9.7)	8 (9.0)	4 (16.7)

*CRS* cytokine release syndrome, *ICANS* immune effector cell-associated neurotoxicity syndrome, *IMWG-FP* International Myeloma Working Group Frailty Proxy, *IQR* interquartile range, *PML* progressive multifocal leukoencephalopathy, *PRFP* patient-reported frailty phenotype, *SFS* Simplified Frailty Score, *TEAE* treatment-emergent adverse events.

^a^Chronic kidney disease, respiratory failure, encephalopathy, and pancreatic adenocarcinoma (*n* = 4, one each) resulted in death. The event of encephalopathy was assessed as related to linvoseltamab treatment by the investigator.

^b^The infections resulting in death were COVID-19 pneumonia (*n* = 3), PML (*n* = 2), septic shock (*n* = 2), pneumonia due to *Pneumocystis jirovecii* infection, pneumonia due to influenza infection, infection (*n* = 3, one each) and sepsis due to *Pseudomonas*, *Haemophilus* or *Escherichia* infections (*n* = 3, one each). The two events of PML, the event of pneumonia due to *Pneumocystis jirovecii* infection, and the event of sepsis due to *Pseudomonas* infection, were assessed as related to linvoseltamab treatment by the investigator.

All patients experienced at least one treatment-emergent AE (TEAE). Similar incidences of Grade 3/4 TEAEs (range: 70.8–75.0%) and of serious TEAEs (range: 76.4–80.6%) were observed between non-frail and frail groups, regardless of frailty measure (Table 2). The most common TEAEs (≥20% of patients) are described in Supplementary Table 1. While incidence and severity of most TEAEs were generally similar across non-frail and frail groups by IMWG-FP and SFS, there were differences in certain TEAEs, including, but not limited to, incidences of headache and Grade 3/4 neutropenia, which were lower in frail groups vs non-frail groups. In contrast, for PRFP, some TEAEs such as CRS and pneumonia were more common and/or more severe in the frail groups.

Rates of treatment discontinuation due to TEAEs were generally higher in the non-frail versus frail groups as measured by IMWG-FP (23.4% vs 12.5%) and by SFS (23.3% vs 12.9%) but were similar between the non-frail and frail groups by PRFP (19.1% versus 20.8%; Table 2). Consistently across the 3 measures, frail groups experienced a modestly higher incidence of TEAEs resulting in death (IMWG-FP: 21.9%; SFS: 19.4%; PRFP: 20.8%) versus non-frail groups (IMWG-FP: 11.7%; SFS: 12.8%; PRFP: 12.4%). The incidence of deaths up to 30 days from the end of treatment was low overall, similar across frail versus non-frail patients by PRFP but slightly higher in frail patients by IMWG-FP and SFS. These deaths were due to either adverse events or progressive/recurring disease. Besides infections, which were the most common TEAEs resulting in death up to data cut-off, chronic kidney disease, respiratory failure, encephalopathy (assessed as related to linvoseltamab treatment), and pancreatic adenocarcinoma resulted in the death of four patients.

The incidence of CRS of any grade was similar between non-frail and frail groups per IMWG-FP (45.5% vs 46.9%, respectively) and SFS (46.5% vs 45.2%, respectively), while CRS occurred more frequently in the frail group (58.3%) versus the non-frail group (41.6%) per PRFP (Table 2). One patient experienced Grade 3/4 CRS; this patient was categorized as non-frail by both IMWG-FP and SFS but frail by PRFP. No CRS events led to treatment discontinuations or death. CRS management and supportive measures used for single CRS events in frail and non-frail groups are described in Supplementary Table 2. The incidence of ICANS was low (8% overall, *n* = 9/117) [26] and largely similar across frail groups (IMWG-FP: 12.5%; SFS: 9.7%; PRFP: 8.3%) and non-frail groups (IMWG-FP: 5.2%; SFS: 7.0%; PRFP: 7.9%), with these variations in proportions being due to differences in only one or three patients within each group (Table 2). Grade 3/4 ICANS was reported in three patients, of whom two were categorized as frail by both IMWG-FP and PRFP, while only one was categorized as frail per SFS. No ICANS events led to death, dose reductions, or treatment discontinuations in any of the three frail groups. The incidence of Grade 3/4 infections was also similar in non-frail (IMWG-FP: 35.1%; SFS: 36.0%; PRFP: 36.0%) groups and largely similar in frail (IMWG-FP: 40.6%; SFS: 38.7%; PRFP: 41.7%) groups, with the number of patients discontinuing treatment due to infections being low across frail groups (IMWG-FP: 3.1%, *n* = 1; SFS: 3.2%, *n* = 1; PRFP: 12.5%, *n* = 3; Table 2). The infections resulting in death were COVID-19 pneumonia (*n* = 3), progressive multifocal leukoencephalopathy (PML; *n* = 2; assessed as related to linvoseltamab treatment), septic shock (*n* = 2), pneumonia due to *Pneumocystis jirovecii* infection (assessed as related to linvoseltamab treatment), pneumonia due to influenza infection, infection (*n* = 3, one each) and sepsis due to *Pseudomonas* (assessed as related to linvoseltamab treatment), *Haemophilus* or *Escherichia* infections (*n* = 3, one each).

## Discussion

Frailty is internationally recognized in treatment guidelines as an important consideration when selecting treatment for MM [30, 31]. However, despite the availability of validated frailty indices, clinical-trial outcomes are not consistently stratified by frailty status, limiting their relevance in guiding evidence-based treatment decisions in this patient subgroup with typically poor prognosis.

Current guidelines from the US National Comprehensive Cancer Network® (NCCN®) recommend to assess older adults for frailty, as well as consider dose modifications based on functional status and age [31]. In view of the recent advances in the MM treatment landscape, it is important to carefully assess the risk–benefit profile of newer therapies like bsAbs in patients meeting frailty criteria, for which there is a paucity of data; in fact, there are limited analyses on efficacy and safety outcomes of other BCMA-directed bispecific antibodies in frail and non-frail populations, and only utilizing the SFS scale. For example, the ORR with elranatamab from the MagnetisMM-3 study was slightly higher in the non-frail group (63.1% [95% CI 51.9–73.4]) than in the frail group (56.4% [95% CI 39.6–72.2]) for patients who were categorized per SFS, but DOR and probabilities of maintaining a response at 12 months were similar between groups (76.0% in non-frail and 70.5% in the frail group) [32]. In a retrospective analysis of teclistamab, patients with MM aged ≥70 years categorized as frail per SFS had a higher ORR (66%) compared with patients with higher physical fitness (50%), while showing similar 12-month OS (61.5% and 64.9%, respectively) [21]. Neither of the two studies above showed markedly increased rates of AEs among patients categorized as frail versus non-frail, but higher risks of Grade ≥2 CRS, Grade ≥2 ICANS, and Grade ≥3 infections were observed in the frail group receiving teclistamab [21, 32].

In this *post hoc* analysis from the LINKER-MM1 study in patients with RRMM, linvoseltamab was evaluated using three frailty measures, thus making this the most comprehensive frailty analysis for bsAbs. Although patients with significant comorbidities were excluded from the study, an appreciable proportion of the LINKER-MM1 population included patients who presented with characteristics of increased frailty, with BMPC ≥60% being more prevalent in frail versus non-frail patients by IMWG-FP and SFS, and by IMWG-FP and PRFP, respectively. Similarly, the presence of EMP per IRC and sBCMA ≥400 ng/mL was more prevalent in frail versus non-frail patients across all measures. Reflecting the contribution of age to categorization by IMWG-FP or SFS, frail groups were generally older than the corresponding non-frail groups. Proportions of patients with MM with ISS and R-ISS stage III were higher per IMWG-FP and SFS in frail versus non-frail groups, in line with previous studies suggesting that ISS is a prognostic marker in older patients [33, 34]. Generally, frail groups tended to have higher rates of high-risk factors, but differences between the components of the frailty scoring measures led to differences in the prevalence of each high-risk factor across frail groups. Across all three frailty measures, frail groups exhibited worse baseline HRQOL than non-frail groups; as expected, given that HRQOL-based components directly contributed to the classification of frailty for the PRFP measure, patients categorized as frail by PRFP had lower baseline HRQOL scores than frail groups defined by the other two measures. Efficacy of linvoseltamab 200 mg was generally consistent across frail and non-frail groups per IMWG-FP, SFS, and PRFP frailty measures. In fact, in this analysis, ORRs to treatment with linvoseltamab were generally high across the three measures among frail and non-frail groups (62.5–74.2% and 69.8–73.0%, respectively), and these responses were consistent with other studies assessing bsAbs in patients with RRMM (56–66% frail; 50–63% non-frail) [21, 32]. Furthermore, the favorable outcomes from linvoseltamab use were evident among frail patients as well as non-frail patients in this clinical study, as demonstrated by the efficacy data in these patients. With a median OS approaching the 2-year mark in both frail and non-frail groups, these results show the efficacy resulting from prolonged treatment with linvoseltamab. However, it is important to note that the limited number of non-frail and frail patients at risk in the KM analysis may have impacted the observation with respect to the numerical differences in DOR and PFS beyond the 12-month time point. In this *post hoc* analysis, efficacy outcomes remained generally consistent across frail and non-frail groups, with the modest variations found for patient baseline characteristics due to the differing components influencing patient categorization by each frailty measure, as well as for some PFS and OS rates.

Overall, the safety profile of linvoseltamab in frail groups across all frailty measures was generally consistent with that of the overall LINKER-MM1 population [2]. In fact, safety and tolerability, including rates of Grade 3/4 and serious TEAEs, were largely similar in frail and non-frail groups across all frailty measures, suggesting that linvoseltamab may have a similar risk–benefit profile across frail and non-frail groups in this study. This observation must be taken into the context of the LINKER-MM1 study population, which excluded patients with an ECOG-PS ≥2 and who had other significant comorbidities [2, 26]. It is also important to note that any differences in response rates or TEAE incidence may be confounded by the small number of patients within each group. Although serious TEAEs were similar between frail and non-frail patients, lower proportions of patients categorized as frail per IMWG-FP or SFS discontinued treatment due to TEAEs than those categorized as non-frail (12.5% and 12.9% versus 23.4% and 23.3%, by each measure); discontinuation rates were similar across the two groups by PRFP. Conversely, the incidence of TEAEs resulting in death was slightly higher in frail versus non-frail patients across all frailty measures; however, it is reasonable to note that even a difference in two or three events may produce a large percent difference within small patient samples, such as the frail sample groups in this analysis. Of all the TEAEs resulting in death (*n* = 17), a total of five events, four due to infections and one encephalopathy, were assessed as related to linvoseltamab treatment.

Commonly reported TEAEs associated with T-cell–engaging therapies include CRS, neurotoxicity, and infections [2, 19, 20]. In the present study, CRS, ICANS, and infections were mostly Grade 1/2; a few of these events led to the discontinuation of linvoseltamab: in the entire population, there were no discontinuations due to CRS, while there was one (0.9%) discontinuation due to ICANS, and 12 (10.3%) discontinuations due to infections. While there were some variations in event rates between frail and non-frail groups, these differences were not consistent across the three frailty measures; most TEAEs reported were not more common in frail versus non-frail patients and no consistent TEAE patterns could be determined across the frailty measures.

Retrospective analyses such as this one may have several limitations. Firstly, the lack of a complete set of baseline assessments may have prevented categorization of all patients by frailty measures. In this specific analysis, a few patients (>10.5%) could not be categorized by all frailty measures due to missing scores. Moreover, as this study evaluated outcomes and frailty in a clinical-trial setting, the impact of compounding comorbidities that would have made patients ineligible for the trial (for example “ultra-frail” patients, those who are aged >80 years, or aged ≤80 years with an IMWG index score ≥3, significant comorbidities and/or impairments in activities of daily living) remains to be ascertained [35–37]. The LINKER-MM1 study was not designed or powered for a prospective frailty analysis, meaning that formal hypothesis testing was not performed. Finally, small sample sizes in frail groups, especially after 12 months, may limit the interpretability of long-term safety and efficacy outcomes.

In conclusion, the LINKER-MM1 study patients who met the criteria for frailty tended to have high rates of high-risk features such as R-ISS stage III disease, high levels of BMPC, EMP, and sBCMA. With three different frailty measures – IMWG-FP, SFS, and PRFP—linvoseltamab showed a risk–benefit profile that was similarly favorable in frail and non-frail groups in the context of the RRMM LINKER-MM1 study population [2]. Continued evaluation of linvoseltamab in frail patients with RRMM in clinical trials and real-world settings is warranted.

## Supplementary information

Supplement.

## Data Availability

Qualified researchers may request access to study documents (including the clinical study report, study protocol with any amendments, blank case report form, statistical analysis plan). Individual anonymized participant data will be considered for sharing: 1) once the product and indication has been approved by major health authorities (e.g., FDA, EMA, PMDA, etc.) or development of the product has been discontinued globally for all indications on or after April 2020 and there are no plans for future development, 2) if there is legal authority to share the data, and 3) there is not a reasonable likelihood of participant re-identification. Submit requests to https://vivli.org/.
