# Supplementary material for: Linvoseltamab outcomes by frailty status in patients with relapsed/refractory multiple myeloma: a subgroup analysis of the LINKER-MM1 study
Source: Blood Cancer J. 2026 Jul 25;16(1):170. doi: 10.1038/s41408-026-01572-1 (PMC13633265; doi:10.1038/s41408-026-01572-1)
Supplement: Supplementary file 1 [file 41408_2026_1572_MOESM1_ESM.pdf]

SUPPLEMENTARY MATERIAL

Linvoseltamab outcomes by frailty status in patients with relapsed/refractory multiple myeloma: A subgroup analysis of the LINKER-MM1 study

Authors: Hira Mian, Naresh Bumma, Joshua Richter, Mina Awad, Timothy J. Inocencio, C. Akunna Otele, Megan Seraphin, Tito Roccia, Glenn S. Kroog, Tanya M. Wildes

Table of contents

SUPPLEMENTARY METHODS 2

Patient-reported outcome assessments 2

SUPPLEMENTARY MATERIALS 3

Supplementary Table 1. TEAEs in ≥20% of patients 3

Supplementary Table 2. CRS management 5

Supplementary Fig. 1 The components of the three frailty measures. 6

SUPPLEMENTARY REFERENCES 7

SUPPLEMENTARY METHODS

Patient-reported outcome assessments

Patient-reported outcome assessments will include generic and disease-specific HRQOL instruments that are commonly used in cancer populations, including the EORTC QLQ-C30 and EuroQoL five dimensions 3-level version.

The EORTC QLQ-C30 is a 30-item, generic questionnaire used to assess HRQOL in patients with cancer [1,2]. It is one of the most frequently used generic measures of HRQOL in cancer clinical trials. The EORTC QLQ-C30 assesses HRQOL across multiple domains, including global health status, global quality of life, functioning (physical, role, emotional, cognitive, and social functioning), symptom scales (fatigue, nausea and vomiting, pain), and single items (dyspnea, insomnia, constipation, diarrhea, financial impact, and appetite loss).

The EuroQoL five dimensions 3-level version is a self-administered generic standardized health status measure, consisting of an EuroQoL five dimensions descriptive system and a EuroQoL visual analog scale. The EuroQoL five dimensions 3-level version descriptive system assesses five dimensions of health: mobility, self-care, usual activities, pain/discomfort, and anxiety/depression. Each dimension is rated on a three-level scale: no problems, some problems, and extreme problems. The EuroQoL visual analog scale component is a vertical visual analog scale used by patients to rate their health.

Patients will be asked to complete the questionnaires prior to any study-related procedures, treatment, or clinician assessment in order to minimize the influence of these external variables on the patient self-report of these PRO measures.

Non-English-speaking patients will complete the questionnaire using validated language translations developed and recommended for each instrument.

SUPPLEMENTARY MATERIALS

Supplementary Table 1. TEAEs in ≥20% of patients

	IMWG-FP				SFS				PRFP			
	Non-frail (*n* = 77)		Frail (*n* = 32)		Non-frail (*n* = 86)		Frail (*n* = 31)		Non-frail (*n* = 89)		Frail (*n* = 24)	
**TEAEs, n (%)**	**Any grade**	**Grade 3/4**	**Any grade**	**Grade 3/4**	**Any grade**	**Grade 3/4**	**Any grade**	**Grade 3/4**	**Any grade**	**Grade 3/4**	**Any grade**	**Grade 3/4**
Number of patients with TEAE	77 (100)	56 (72.7)	32 (100)	24 (75.0)	86 (100)	63 (73.3)	31 (100)	23 (74.2)	89 (100)	66 (74.2)	24 (100)	17 (70.8)
CRS	35 (45.5)	1 (1.3)	15 (46.9)	0	40 (46.5)	1 (1.2)	14 (45.2)	0	37 (41.6)	0	14 (58.3)	1 (4.2)
Neutropenia[a]	37 (48.1)	37 (48.1)	12 (37.5)	11 (34.4)	41 (47.7)	41 (47.7)	10 (32.3)	9 (29.0)	38 (42.7)	37 (41.6)	12 (50.0)	12 (50.0)
Diarrhea	33 (42.9)	2 (2.6)	14 (43.8)	0	35 (40.7)	2 (2.3)	14 (45.2)	0	36 (40.4)	0	12 (50.0)	2 (8.3)
Anemia[a]	31 (40.3)	22 (28.6)	14 (43.8)	12 (37.5)	35 (40.7)	25 (29.1)	12 (38.7)	11 (35.5)	36 (40.4)	27 (30.3)	10 (41.7)	8 (33.3)
Cough	30 (39.0)	0	11 (34.4)	0	35 (40.7)	0	12 (38.7)	0	38 (42.7)	0	7 (29.2)	0
Fatigue	26 (33.8)	0	12 (37.5)	1 (3.1)	29 (33.7)	1 (1.2)	11 (35.5)	0	32 (36.0)	0	7 (29.2)	1 (4.2)
Arthralgia	24 (31.2)	1 (1.3)	11 (34.4)	1 (3.1)	29 (33.7)	1 (1.2)	9 (29.0)	1 (3.2)	28 (31.5)	0	10 (41.7)	2 (8.3)
Headache[a]	25 (32.5)	1 (1.3)	3 (9.4)	0	28 (32.6)	1 (1.2)	2 (6.5)	0	26 (29.2)	1 (1.1)	4 (16.7)	0
Nausea	21 (27.3)	0	7 (21.9)	0	20 (23.3)	0	8 (25.8)	0	24 (27.0)	0	4 (16.7)	0
Dyspnea	20 (26.0)	1 (1.3)	7 (21.9)	0	20 (23.3)	0	7 (22.6)	1 (3.2)	25 (28.1)	1 (1.1)	2 (8.3)	0
Hypokalemia[a]	20 (26.0)	2 (2.6)	7 (21.9)	2 (6.3)	22 (25.6)	2 (2.3)	7 (22.6)	2 (6.5)	22 (24.7)	2 (2.2)	6 (25.0)	2 (8.3)
Vomiting	19 (24.7)	0	6 (18.8)	0	19 (22.1)	0	6 (19.4)	0	23 (25.8)	0	2 (8.3)	0
Back pain	19 (24.7)	2 (2.6)	5 (15.6)	1 (3.1)	20 (23.3)	2 (2.3)	7 (22.6)	1 (3.2)	20 (22.5)	1 (1.1)	6 (25.0)	2 (8.3)

COVID-19[a,b]	17 (22.1)	8 (10.4)	7 (21.9)	2 (6.3)	19 (22.1)	9 (10.5)	7 (22.6)	2 (6.5)	19 (21.3)	8 (9.0)	5 (20.8)	2 (8.3)
Upper respiratory tract infection	17 (22.1)	1 (1.3)	7 (21.9)	1 (3.1)	18 (20.9)	1 (1.2)	7 (22.6)	1 (3.2)	20 (22.5)	2 (2.2)	5 (20.8)	0
Thrombocytopenia[a]	16 (20.8)	13 (16.9)	7 (21.9)	4 (12.5)	17 (19.8)	13 (15.1)	6 (19.4)	4 (12.9)	18 (20.2)	12 (13.5)	5 (20.8)	5 (20.8)
Edema peripheral	13 (16.9)	1 (1.3)	7 (21.9)	1 (3.1)	12 (14.0)	0	9 (29.0)	2 (6.5)	17 (19.1)	1 (1.1)	4 (16.7)	1 (4.2)
Hypogammaglobulinemia	16 (20.8)	1 (1.3)	3 (9.4)	0	15 (17.4)	1 (1.2)	5 (16.1)	0	17 (19.1)	1 (1.1)	3 (12.5)	0
Pneumonia	12 (15.6)	11 (14.3)	7 (21.9)	6 (18.8)	17 (19.8)	15 (17.4)	4 (12.9)	4 (12.9)	11 (12.4)	10 (11.2)	9 (37.5)	8 (33.3)
Constipation	11 (14.3)	0	10 (31.3)	0	14 (16.3)	0	7 (22.6)	0	17 (19.1)	0	4 (16.7)	0
Pyrexia	15 (19.5)	0	6 (18.8)	0	17 (19.8)	0	4 (12.9)	0	15 (16.9)	0	6 (25.0)	0
UTI	7 (9.1)	1 (1.3)	4 (12.5)	1 (3.1)	9 (10.5)	2 (2.3)	4 (12.9)	0	7 (7.9)	1 (1.1)	5 (20.8)	1 (4.2)

[a]Composite terms. [b]A total of three patients experienced Grade 5 COVID-19 events, who were categorized as non-frail or frail per IMWG-FP (non-frail: *n* = 2, 2.6%; frail: *n* = 1, 3.1%), per SFS (non-frail: *n* = 2, 2.3%; frail: *n* = 1, 3.1%), and per PRFP (non-frail: *n* = 1, 1.1%; frail: *n* = 2, 8.3%).

CRS, cytokine release syndrome; IMWG-FP, International Myeloma Working Group Frailty Proxy; PRFP, patient-reported frailty phenotype; SFS, Simplified Frailty Score; TEAE, treatment-emergent adverse event; UTI, urinary tract infection.

Supplementary Table 2. CRS management

Number of patients with supportive measures for single CRS event, n (%)	IMWG-FP		SFS		PRFP	
	**Non-frail (*n* = 77)**	**Frail (*n* = 32)**	**Non-frail (*n* = 86)**	**Frail (*n* = 31)**	**Non-frail (*n* = 89)**	**Frail (*n* = 24)**
Tocilizumab only	8 (10.4)	4 (12.5)	12 (14.0)	3 (9.7)	10 (11.2)	3 (12.5)
Corticosteroid only	5 (6.5)	1 (3.1)	4 (4.7)	2 (6.5)	4 (4.5)	2 (8.3)
Concurrent tocilizumab and corticosteroids	3 (3.9)	2 (6.3)	5 (5.8)	1 (3.2)	3 (3.4)	3 (12.5)
Both tocilizumab and corticosteroids, not concurrent	2 (2.6)	0	2 (2.3)	0	1 (1.1)	1 (4.2)

CRS, cytokine release syndrome; IMWG-FP, International Myeloma Working Group Frailty Proxy; PRFP, patient-reported frailty phenotype; SFS, Simplified Frailty Score.

Supplementary Fig. 1 The components of the three frailty measures.

IMWG-FP score

Original IMWG-FI Variables [3]	IMWG-FP[a]	Score
**Age**<br>• ≤75<br>• 76–80<br>• >80	**Age**<br>• ≤75<br>• 76–80<br>• >80	0<br>1<br>2
**CCI**<br>• ≤1<br>• ≥2	**CCI**<br>• ≤1<br>• ≥2	0<br>1
**ADL**<br>• >4<br>• ≤4	**EQ-5D-3L: self-care score**<br>• No problems<br>• Moderate / severe	0<br>1
**IADL**<br>• >5<br>• ≤5	**EQ-5D-3L: usual activities score**<br>• No problems<br>• Moderate / severe	0<br>1
	**Sum of scores**<br>• Non-frail<br>• Frail	0/1<br>≥2

SFS score

Original IMWG-FI Variables [3]	SFS [4]	Score
**Age**<br>• ≤75<br>• 76–80<br>• >80	**Age**<br>• ≤75<br>• 76–80<br>• >80	0<br>1<br>2
**CCI**<br>• ≤1<br>• ≥2	**CCI**<br>• ≤1<br>• ≥2	0<br>1
**ADL**<br>• >4<br>• ≤4<br>**IADL**<br>• >5<br>• ≤5	**ECOG PS**<br>• 0<br>• 1<br>• ≥2	0<br>1<br>2
	**Sum of scores**<br>• Non-frail<br>• Frail	0/1<br>≥2

PRFP score

Fried Frailty Criteria [5]	EORTC QLQ-C30 Candidate Item for PRFP[b]	Score
Unintentional weight loss (≥10 pounds or ≥5% of body weight within the past year)	Have you lacked appetite? (Q13)<br>• Not at all/a little<br>• Quite a bit/very much	0<br>1
Exhaustion (self-reported)	Did you need to rest? (Q10)<br>• Not at all/a little<br>• Quite a bit/very much	0<br>1
Weakness (typically measured by grip strength)	Have you felt weak? (Q12)<br>• Not at all/a little<br>• Quite a bit/very much	0<br>1
Slowness (typically measured by observing gait speed)	Do you have any trouble taking a short walk outside of the house? (Q3)<br>• Not at all/a little<br>• Quite a bit/very much	0<br>1
Low physical activity (typically measured by Kcals expended per week using the short version of the Minnesota Leisure Time Activity Questionnaire)	Were you limited in doing either your work or other daily activities? (Q6)<br>• Not at all/a little<br>• Quite a bit/very much	0<br>1
	**Sum of scores**<br>• Non-frail<br>• Frail	0–2<br>≥3

[a]Modified IMWG-FP score; the original IMWG-FP used EQ-5D-3L and EQ-5D-5L, and categorized patients as fit, intermediate fit, or frail [6,7]; [b]The original PRFP categorized patients as fit, pre-frail, or frail [7,8].

ADL, Activity of Daily Living; CCI, Charlson Comorbidity Index; ECOG-PS, Eastern Cooperative Oncology Group performance status; SFSEORTC QLQ-C30, European Organization for Research and Treatment of Cancer Quality of Life 30-item Questionnaire; EQ-5D-3L, EuroQoL five dimensions 3-level version; EQ-5D-5L, EuroQoL five dimensions 5-level version; IADL, Instrumental Activity of Daily Living; IMWG, International Myeloma Working Group; IMWG-FI, International Myeloma Working Group Frailty Index; IMWG-FP, IMWG Frailty Proxy; PRFP, patient-reported frailty phenotype; SFS, Simplified Frailty Proxy.

SUPPLEMENTARY REFERENCES

1. Cocks K, Cohen D, Wisloff F, Sezer O, Lee S, Hippe E, et al. An international field study of the reliability and validity of a disease-specific questionnaire module (the QLQ-MY20) in assessing the quality of life of patients with multiple myeloma. Eur J Cancer. 2007; 43:1670–167.
2. Stead ML, Brown JM, Velikova G, Kaasa S, Wisloff F, Child JA, et al. Development of an EORTC questionnaire module to be used in health-related quality-of-life assessment for patients with multiple myeloma. European Organization for Research and Treatment of Cancer Study Group on Quality of Life. Br J Haematol. 1999; 104:605–611.
3. Palumbo A, Bringhen S, Mateos MV, Larocca A, Facon T, Kumar SK, et al. Geriatric assessment predicts survival and toxicities in elderly myeloma patients: an International Myeloma Working Group report. Blood 2015; 125:2068–2074.
4. Facon T, Dimopoulos MA, Meuleman N, Belch A, Mohty M, Chen WM, et al. A simplified frailty scale predicts outcomes in transplant-ineligible patients with newly diagnosed multiple myeloma treated in the FIRST (MM-020) trial. Leukemia. 2020; 34:224–233.
5. Fried LP, Tangen CM, Walston J, Newman AB, Hirsch C, Gottdiener et al. Frailty in Older Adults: Evidence for a Phenotype. J Gerontol A Biol Sci Med Sci. 2001; 56:M146–M157.
6. Facon T, Hulin C, Dimopoulos MA, Belch A, Meuleman N, Mohty M, et al. A Frailty Scale Predicts Outcomes of Patients with Newly Diagnosed Multiple Myeloma Who Are Ineligible for Transplant Treated with Continuous Lenalidomide Plus Low-Dose Dexamethasone on the First Trial. Blood. 2015; 126:4239.
7. Murugappan MN, King-Kallimanis BL, Bhatnagar V, Kanapuru B, Farley JF, Seifert RD, et al. Patient-reported frailty phenotype (PRFP) vs. International Myeloma Working Group frailty index (IMWG FI) proxy: A comparison between two approaches to measuring frailty. J Geriatr Oncol 2024; 15:101681.

8. Murugappan MN, King-Kallimanis BL, Bhatnagar V, Kanapuru B, Farley JF, Seifert RD, et al. Measuring Frailty Using Patient-Reported Outcomes (PRO) Data: A Feasibility Study in Patients with Multiple Myeloma. Qual Life Res 2023; 32:2281–2292.
